# T cells in Group A *Streptococcus* infection: protection or pathology?

**DOI:** 10.3389/fcimb.2026.1876394

**Published:** 2026-09-17

**Authors:** Liya Mathew, Mohammad Ali, Hermaleigh Townsley, Alice Halliday, Paul Klenerman, Thushan I. de Silva, Anu Goenka

**Affiliations:** 1 Biochemistry and Biomedical Sciences, Biomedical Sciences Building, University of Bristol, Bristol, United Kingdom; 2 Bristol Vaccine Centre, Bristol Medical School, University of Bristol, Bristol, United Kingdom; 3 National Institute for Health and Care Research (NIHR) Health Protection Research Focus Award in Vaccines and Immunisation, University of Bristol, Bristol, United Kingdom; 4 Peter Medawar Building for Pathogen Research, Nuffield Department of Medicine, University of Oxford, Oxford, United Kingdom; 5 Department of Biochemistry and Molecular Biology, Bangladesh Medical University, Shahbag, Dhaka, Bangladesh; 6 The Florey Institute of Infection, University of Sheffield, Sheffield, United Kingdom; 7 National Institute for Health and Care Research (NIHR) Sheffield Biomedical Research Centre, University of Sheffield, Sheffield, United Kingdom; 8 Division of Clinical Medicine, School of Medicine and Population Health, University of Sheffield, Sheffield, United Kingdom; 9 Translational Gastroenterology and Liver Unit, John Radcliffe Hospital, Oxford, United Kingdom; 10 National Institute for Health and Care Research (NIHR) Oxford Biomedical Research Centre, University of Oxford, Oxford, United Kingdom; 11 Vaccines and Immunity Theme, Medical Research Council (MRC) Unit The Gambia at the London School of Hygiene & Tropical Medicine, Fajara, Gambia; 12 Faculty of Infectious and Tropical Disease, London School of Hygiene & Tropical Medicine, London, United Kingdom; 13 Paediatric Immunology and Infectious Diseases, Bristol Royal Hospital for Children, Bristol, United Kingdom

**Keywords:** correlates of protection, GAS, *Streptococcus pyogenes*, T cells, vaccines

## Abstract

*Streptococcus pyogenes* (Group A *Streptococcus*; GAS) is a World Health Organization (WHO) priority pathogen for vaccine development. GAS vaccine development and trials have been impeded by a poor understanding of GAS immunity, and to date no correlate of protection has been identified. The predominant focus of efforts has been to establish antibody correlates of protection. However, T cell immunity may provide important contributions to clinical protection from GAS. This is supported by a possible relationship between the life-course ontogeny of T cell reactivity and repertoire compared with age-related GAS epidemiology. T cells are also the target of superantigens, a key GAS virulence mechanism. Furthermore, T cells have important functions in protection against other extracellular respiratory bacteria. Here, we explore immune risk factors associated with GAS epidemiology and clinical manifestations highlighting the dual protective (e.g. GAS-specific Th17 cells) and pathogenic (e.g. CD8+ T cells) role of T cells in GAS infection and how this could be harnessed in future research.

## 1 Introduction

*Streptococcus pyogenes* (Group A *Streptococcus*; GAS) has been identified by the World Health Organization (WHO) as a priority pathogen for vaccine development (Hasso-Agopsowicz et al., 2024). It causes over half a million deaths annually, particularly in high-burden GAS settings (e.g. low- and middle-income countries) among individuals experiencing socioeconomic deprivation, overcrowding and limited access to healthcare (Nelson et al., 2016). GAS infection is associated with a wide spectrum of clinical manifestations including superficial, invasive and post-infectious immune-mediated sequelae (Hand et al., 2020). An important and consistent observation is that the incidence of GAS infection decreases following a peak in childhood years, suggesting that clinical protection from natural infection is possible (Tsoi et al., 2015).

Vaccine development and vaccine trials have been impeded by a lack of understanding of protective immunity to GAS infection. Most GAS immunity studies to date have focused on antibody responses. However, T cell-dependent antibody production contributes to clinical protection against extracellular bacteria (Malkiel et al., 2000; Yates et al., 2013). Thus, elucidating clinically important protective T cell responses will be fundamental to discovering GAS correlates of protection and directing vaccine antigen discovery. Furthermore, T cells are profoundly subverted by superantigens, an exotoxin armoury of virulence factors that have played a key role in successful global propagation of some GAS strains, such as M1_UK_ (Vieira et al., 2024). However, T cell immunity may be partially redundant and pleiotropic given that clinical T cell immunodeficiencies have not been associated with increased risk of GAS infection. Here, we review existing data on the role of T cells in GAS infection and their possible contribution to protective immunity.

## 2 Age-related epidemiology of GAS and life-course T cell ontogeny

The age-related epidemiology of different GAS infection phenotypes offers potential insights into the role of T cells in protective immunity. Broadly, transplacental IgG wanes during the first 6 months of life and infants become increasingly vulnerable to pathogens such as respiratory syncytial virus (RSV) and pathobionts such as *Streptococcus pneumoniae* (Cannon et al., 2021). Invasive GAS infection is most prevalent amongst infants under 2 years of age, and adults over 65 years of age (**Figure 1A**) (Nelson et al., 2016). However, pharyngeal GAS infection appears less common during infancy. GAS pharyngitis consistently exhibits an epidemiological peak during school-age years, before the incidence falls significantly in late adolescence and adulthood (**Figure 1A**) (Cannon et al., 2021). Pyoderma is a superficial skin manifestation of GAS infection that is prevalent in low-income settings. In these contexts, the incidence of pyoderma peaks amongst children 1–5 years of age and is often associated with preceding minor skin trauma/abrasion (Armitage et al., 2019). While these epidemiological observations may be influenced by exposure/contact patterns (particularly for pyoderma), they suggest that waning of transplacental antibody alone is not sufficient to create a window of vulnerability to invasive GAS in the first year of life. A reduced incidence of almost all GAS infection phenotypes between the second and sixth decades of life suggests that through successive exposure, perhaps to different GAS serotypes, natural immunity is possible (**Figure 1A**) (Tsoi et al., 2015).

**Figure 1:**
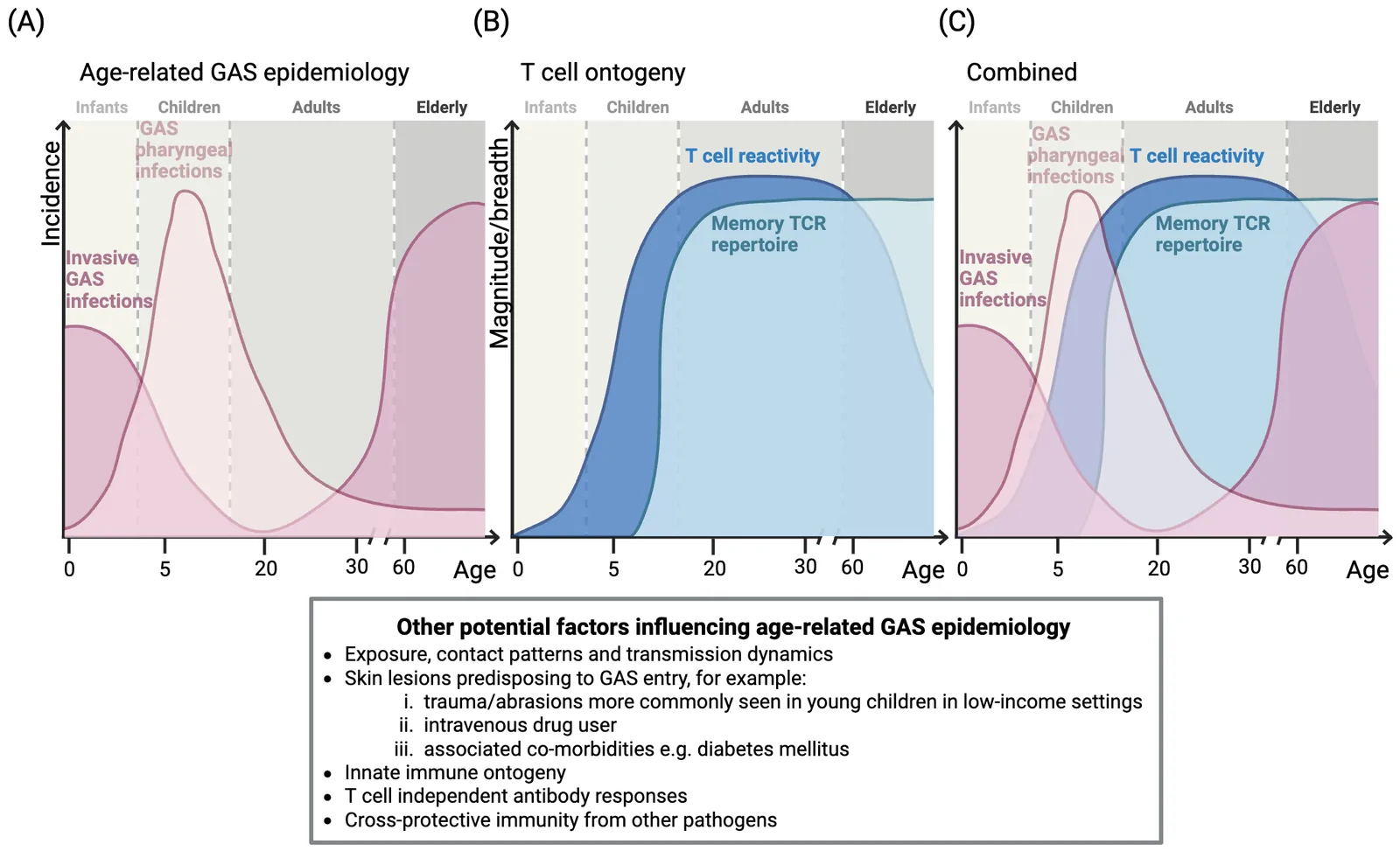
The relationship between age-related GAS epidemiology and T cell ontogeny. **(A)** Life course GAS infection incidence where dark pink line shows invasive GAS and light pink line shows pharyngeal GAS infections. **(B)** Life course T cell responses where light blue line shows memory T cell repertoire and dark blue line show T cell ‘reactivity’ to mitogen stimulation. **(C)** Relationship between age-related GAS epidemiology and T cell ontogeny. Created in BioRender.

Our understanding of early life T cell ontogeny may offer insights into GAS-specific natural immunity. Infant T cell responses are adapted to tolerance. For example, regulatory T cells (Tregs) account for approximately 10% of total CD4+ T cells in infant tissue compared to ~5% in adult tissue (Thome et al., 2016). In addition, mucosal memory T cells before the age of 3 years exhibit reduced ability to produce proinflammatory cytokines, such as IFN-γ and TNF, compared to older children (Connors et al., 2023). This manifests as reduced *in vitro* ‘reactivity’ to mitogen stimulation (**Figure 1B**) (Rudolph et al., 2018). It is unknown whether reduced infant T cell reactivity could be somehow biologically linked with reduced pharyngeal GAS infection during early life.

The risk of invasive GAS significantly increases beyond the sixth decade of life (**Figure 1A**), coinciding with a period of profound changes affecting almost all elements of the immune system, including reduced T cell ‘reactivity’ (**Figure 1B**), collectively termed immunosenescence. In contrast, the breadth of T cell receptor (TCR) clonality increases sharply during school-age years in memory cells resulting in a wide T cell repertoire that persists throughout adulthood (**Figure 1B**) (Sun et al., 2022). While this seemingly corresponds with an increase in pharyngeal GAS infections, other factors must be considered. Multiple interacting mechanisms may impact the age-related epidemiology of GAS infection including social/environmental determinants, GAS exposure patterns, microbiome factors as well as changes in innate and humoral immunity, including cross-protective responses (Zinkernagel, 2000). Despite this, given these parallels between age-related GAS epidemiology and life course T cell ontogeny (**Figure 1C**), there is a need to characterise GAS-specific T cell responses in different age cohorts experimentally.

## 3 Clinical immunosuppression and GAS

Primary immune disorders (PIDs) offer insights into the non-redundancy of clinically protective immune pathways. However, at present, no PIDs have been systematically associated with an increased risk of GAS infection. This suggests some elements of redundancy in the role of adaptive immune populations, or a more complex pleiotropic role in both pathogenesis and protection. The only known clinical immune susceptibility factor has been observed in patients receiving IL-1β inhibitor therapy who appear to have an approximately 330-fold increased risk of invasive GAS infections, in particular necrotising fasciitis (LaRock et al., 2016). In mice, IL-1β has been shown to enhance expansion of antigen-primed CD4+ and CD8+ T cells, in particular IL-17-producing effector CD4+ T cells (Ben-Sasson et al., 2013). Human *in vitro* studies have shown that IL-1β (and IL-23) are essential drivers of Th17 differentiation (Revu et al., 2018). IL-6 may also be important in human GAS infection, with invasive GAS reported in a patient with a homozygous *IL6ST* mutation, as well as a patient receiving anti-IL-6 immunotherapy (Schwerd et al., 2017; Wilde et al., 2021). Of note, IL-6 deficient mice failed to develop a Th17 response leading to fatal GAS infection within 7 days (Dileepan et al., 2011).

## 4 Other extracellular bacteria and T cell immunity

T cells contribute to immunity against extracellular respiratory bacteria. For example, reduced CD4+ count is associated with susceptibility to *S. pneumoniae* and *Staphylococcus aureus* in HIV-infected patients (Capocci and Lipman, 2013). Furthermore, patients treated with antiretroviral therapy associated with successful T cell reconstitution have a significantly reduced risk of invasive pneumococcal pneumonia (Reddy et al., 2010). Therefore, understanding the role of T cells in immunity to extracellular bacteria (e.g. *S. aureus*, *S. pneumoniae*, and *Pseudomonas aeruginosa*) may help inform understanding of T cells in GAS infection.

A unifying characteristic of such bacteria is their ability to evade the T cell response, particularly through modulation of Th17 cells and Tregs (Neill et al., 2012; Gray et al., 2014). These cells are abundant at mucosal surfaces and share signalling and differentiation pathways determined by environmental and cellular cues (Xie et al., 2026). These processes can be subverted by bacteria for immune evasion through altered cytokine signalling and induction of regulatory pathways.

In murine *P. aeruginosa* infection, the depletion of γδ T cells led to increased bacterial load, associated with a reduction in IL-17, IL-22, IL-23 and RORγT levels (Liu et al., 2013). Similarly with *S. aureus* murine infection, depletion of IL-17 signalling led to increased mortality and bacterial load (Bartsch et al., 2022), highlighting the potentially protective role of Th17 immunity. Significantly, autosomal dominant hyper-IgE syndrome caused by *STAT3* gain-of-function mutation is associated with diminished Th17 cells and susceptibility to *S. aureus*, as well as *S. pneumoniae* (Milner et al., 2008). Th1 immunity has also been implicated in *S. aureus* skin infections, where specific IFN-γ producing CD4+ T cells are expanded following infection in both mice and humans (Brown et al., 2015; Marchitto et al., 2019).

A key finding from murine *S. aureus* skin and soft tissue infection models is the role of prior infection in modulating vaccine-induced cellular immunity. Diminished vaccine efficacy was seen following immunisation with *S. aureus* antigens among mice with prior exposure due to impaired expansion of both effector T cells and dendritic cells (Teymournejad et al., 2023). The protective efficacy was restored when utilising an adjuvant that specifically stimulated Th1/Th17 cell responses (Teymournejad et al., 2023). This emphasises the need to study antigen-specific T cell responses to prior infection when considering GAS vaccine design.

Superantigens and other exotoxins produced by extracellular bacteria play a key role in T cell immunity. Pneumolysin (Ply) is a protein toxin expressed by virtually all pneumococcal strains and is a virulence factor in invasive disease (Berry et al., 1995). Ply and associated toxoids induce IFN-γ producing CD4+ and memory Th17 proliferation in PBMCs and nasal-associated lymphoid tissue from children (Zhang et al., 2007; Gray et al., 2014). Responses were more pronounced in children who were culture negative than those with pneumococcal nasopharyngeal carriage, suggesting that Ply-induced Th17 and CD4+ responses are protective (Zhang et al., 2007; Gray et al., 2014).

## 5 Specific T cell functions in GAS protection and immunity

GAS is a strictly human pathogen with extensive strain diversity affecting multiple tissue microenvironments and causing a plethora of clinical manifestations, many of which cannot be recapitulated in mice. Superantigens and the plasminogen activator streptokinase, key GAS virulence factors, exclusively affect human cells and proteins. This raises challenges when investigating immunity at the laboratory bench where no single model can accurately reproduce the spectrum and complexity of human pathogenesis.

Perhaps unexpectedly, murine models have shown that T cells appear necessary for efficient GAS infection. In mice expressing human HLA, the depletion of CD8+ (but not CD4+) T cells was associated with a lower bacterial burden and reduced inflammatory signature (Zeppa et al., 2017). It was postulated that superantigen may underlie the necessity of an intact CD8+ T cell population for successful GAS infection. In support, this phenomenon is not seen with *S. pneumoniae* murine infection, a pathogen which is not known to express superantigen (Zeppa et al., 2017). These findings also potentially help explain why patients with T cell immunodeficiencies do not appear to be selectively vulnerable to GAS infection, in contrast to the significant vulnerability of these patient groups to invasive pneumococcal disease (Levy et al., 1988; Gennery, 2002).

There is evidence for a variety of T cell phenotypes each contributing both to protection and pathology in GAS infection (**Table 1**). However, as mentioned previously, GAS immunity is complicated by different disease manifestations as well as a variety of infection sites and stages. Future work could focus on how these impact the role of T cells and their ability to be protective or pathogenic. IL-17 immunity represents a promising protective pathway, suggesting the importance of mucosal immunity. In healthy individuals, skin samples showed GAS-responsive T cells were of a Th17 phenotype exhibiting IL-22 production (Chen et al., 2023). IL-17 knockout mice are more susceptible to disseminated GAS infection after intranasal challenge (Mills et al., 2024). Furthermore, increased GAS bacterial burden is observed in the vaginal tract of IL-17A knockout mice (Carey et al., 2016). Severely immunodeficient RAG knockout mice who had lower IL-17A also had less vaginal GAS clearance and greater amounts of proinflammatory cytokines (Carey et al., 2016). RAG mice not only lack T cells, but also B cells. This highlights the difficulty in disentangling the contribution of T cells to clinical protection given their key role in instructing B cells in bacterial immune responses to protein antigens. To add complexity, antibody responses specific to Group A Carbohydrate appear to be at least partly T cell dependent (Fryer et al., 2026). In humans, the importance of Th17-induced IL-17 has been observed in tonsils, which are populated with GAS-specific Th17 cells particularly in individuals with recurrent GAS tonsilitis (Dileepan et al., 2015). These individuals also had lower frequencies of GAS-specific T follicular helper (Tfh) cells and Mucosal-Associated Invariant T (MAIT) cells (Dan et al., 2019; Radcliff et al., 2019) suggesting these could contribute mechanistically to a protective correlate. Despite multiple lines of evidence suggesting the involvement of T cells in protection from GAS infection, different clinical manifestations of GAS could be associated with distinct T cell responses and understanding these will be key to conceptualising vaccines capable of harnessing the protective efficacy of T cells.

**Table 1:** Association of T cell subsets in protection and pathology of GAS infections.

T cell subset	Protective role	Pathological role
CD8+	• Able to lyse GAS-infected cells, so may contribute to control of intracellular GAS (Chen et al., 2023)	• In mice, CD8+ depletion is associated with decreased production of proinflammatory cytokines and lower nasopharyngeal GAS burden (Zeppa et al., 2017)
Treg	• During acute GAS pharyngitis, Treg cell frequency reduces in peripheral blood (Anderson et al., 2022)	• GAS M protein induces a CD4+ regulatory phenotype, producing IL-10 and granzyme B, which can suppress bystander T cells (Price et al., 2005)
Tfh	• Lower germinal centre Tfh frequency is associated with recurrent GAS tonsilitis (Dan et al., 2019) • GAS antigen vaccination led to increased splenic and lymph node Tfh cells in mice (Harbison-Price et al., 2025)	• SpeA can alter Tfh phenotypes, inducing granzyme-B positive ‘killer’ Tfh cells, causing B cell apoptosis and reduced antibody production (Dan et al., 2019)
Th1	• GAS-specific Th1 memory responses appear to contribute to age-related immunity, with robust adult responses compared to childhood (Mortensen et al., 2015) • Vaccines that promote IFN-γ mediated Th1 response in mice are associated with increased protection from invasive disease, independent of antibody response (Rivera-Hernandez et al., 2020)	• Th1 phenotype induced by superantigens and drives excessive cytokine response (Norrby-Teglund et al., 1997) • Implicated in the post-immune sequalae of GAS including RHD and ARF (Kirvan et al., 2022)
Th17	• Lack of Th17 immunity is associated with increased GAS burden in murine mucosal infection (Carey et al., 2016; Mills et al., 2024), and intranasal vaccination induces an antibody-independent Th17 protective response (Fan et al., 2014; Ruiz-Romeu et al., 2016) • Deficiency of IL-6 (a Th17 trophic cytokine) results in fatal GAS infection within 7 days in mice, and human *IL6ST* mutation and anti-IL-6 immunotherapy has been associated with severe GAS (Dileepan et al., 2011; Schwerd et al., 2017; Wilde et al., 2021)	• GAS-specific Th17 response is implicated in post-infection immunological complications such as guttate psoriasis (Ruiz-Romeu et al., 2016) and RHD (Wen et al., 2015; Harbison-Price et al., 2025)
MAIT cells	• Reduced MAIT cells have been observed in recurrent tonsilitis patients (Radcliff et al., 2019) • Can uniquely recognise some GAS antigens (Meermeier et al., 2016)	• MAIT cells driven to superantigen-induced exhaustion could lead to suppressed immunity (Shaler et al., 2017)
iNKT	• Sonicated GAS activates murine iNKT hybridomas to produce IL-2 (Kinjo et al., 2011) • Hypertrophied tonsils in children show high CD1d expression and GAS presence, suggesting iNKT cells as potential regulators of the local immune response (Sarmiento Varón et al., 2021)	
γδ T cells		• Increased frequency of γδTCR^+^ Vδ2^+^ T cells in participants who developed pharyngitis prior to GAS human challenge, and increased activation after challenge (Anderson et al., 2022)

Current understanding of GAS immunity arises largely from murine studies. However, the degree to which murine observations can be extrapolated to humans remains unknown. The Controlled Human Infection for Vaccination Against *S. pyogenes* (CHIVAS) challenge model of pharyngitis has provided a unique opportunity to characterise human immune responses to natural infection (Anderson et al., 2022). The majority of the reported data is related to antibody immunity. In brief, pronounced IgA and minimal IgG responses to key candidate vaccine antigens were relatively associated with protection from pharyngitis (Osowicki et al., 2024). With regards to T cell immunity, prior to infection, γδTCR+Vδ2+ T cells were higher in patients who developed pharyngitis when compared to those who did not (Anderson et al., 2022; Osowicki et al., 2024). Acute infection was associated with a transient reduction in circulating CD4^+^ Th1, Th17, Treg, and Tfh subsets, alongside a reduced CD4^+^ expression of CXCR3, suggesting possible migration of these subsets to the site of infection and therefore a possible role in protection (Anderson et al., 2022). Meanwhile, MAIT and γδTCR^+^ Vδ2^+^ T cells were activated during acute GAS pharyngitis (Anderson et al., 2022). Collectively, these observations suggest that antibody immunity alone may not protect against GAS challenge and highlights the need to better understand the functional roles underpinning the complex dynamics of T cell responses before, during and after human GAS infection.

## 6 The contribution of superantigen in subverting T cell responses

Superantigens are a family of protein exotoxins that act as potent T cell mitogens through excessive and aberrant activation (White et al., 1989). Remarkably, superantigen expression is conserved across all GAS strains, suggesting they may have co-evolved with humans to achieve persistence e.g. asymptomatic colonisation and carriage. Superantigen binds extracellularly to both the MHC class II molecule and T cell receptor Vβ chains, away from conventional peptide-binding sites resulting in activation of up to 25% of peripheral T cells (Choi et al., 1990). Depletion of superantigen-responsive Vβ-specific T cells reduces inflammatory responses *in vivo* (Zeppa et al., 2017). The magnitude of superantigen-induced T cell activation is influenced by the binding strength of the superantigen-HLA class II interaction, which varies with different HLA alleles. After binding, superantigen includes T cell hyperactivation which drives apoptosis, with surviving T cells and MAIT cells exhibiting an anergic phenotype as well as increased proportions of immunomodulatory Tregs (Shaler et al., 2017; Lu et al., 2025), which may contribute to suppressed immunity. Importantly, GAS-specific Tfh cells undergo a phenotypic change when exposed to superantigen, converting to a ‘killer’ phenotype with direct B cell killing, reduced antibody production and reduced chemotactic responses toward CXCL13 (B-cell-attracting chemokine 1) (Dan et al., 2019; Davies et al., 2019).

There is significant variation in the global distribution of GAS serotypes and lineages (Bessen et al., 2018). Furthermore, there are now at least 14 characterized, genetically distinct GAS superantigens, many of which are encoded on bacteriophage elements while some are chromosomally encoded (Beres and Musser, 2007; Shannon et al., 2019). The median number of different superantigen genes expressed by an individual GAS strain is four (Maripuu et al., 2008). Individual superantigen genes vary between lineages, with specific superantigen expression profiles implicated in successful global propagation of some strains, as well as scarlet fever and invasive GAS outbreaks e.g. SpeA (streptococcal pyrogenic exotoxin A) (Dan et al., 2019; Lynskey et al., 2019; Brouwer et al., 2020; Alcolea-Medina et al., 2023). While SpeA has been associated with recent lineages causing invasive disease in the UK, there is geographical variation e.g. SpeC is the most common superantigen found in invasive strains in Pakistan (Khan et al., 2020; Vieira et al., 2024). SpeA is expressed by a wide variety of strains representing the breadth of GAS genomic diversity and six allelic variants have been identified (Nelson et al., 1991; Bessen et al., 1999). *In vitro* analysis found that one allelic variant, SpeA3, had significantly increased mitogenic activity and MHC Class II affinity (Kline and Collins, 1996). However, another superantigen, streptococcal mitogenic exotoxin Z (SmeZ), was found to be 18-fold more potent than SpeA in a lymphocyte proliferation assay (Muller-Alouf et al., 2001). Taken together, this underscores the complexity of the interaction between superantigens and T cells and the impact of both on successful GAS propagation as well as severe disease phenotypes. There is a need to further understand complex molecular, functional and epidemiological interactions between superantigen, HLA class II allele distribution and T cell responses that may play a role in clinical protection.

## 7 The potential role of T cell immunity in GAS vaccine development

GAS vaccine development has been impeded by several factors. Candidate vaccines must not only seek to induce clinical protection across a broad spectrum of GAS strains/serotypes but also avoid inducing autoimmunity e.g. acute rheumatic fever (ARF), glomerulonephritis and other post-infectious conditions. For example, an M protein vaccine administered to 21 siblings of patients with rheumatic fever in the 1960s resulted in some recipients suffering adverse events that were potentially consistent with ARF (Massell, 1969). However, confounding factors have been identified in this study, such as documented GAS infection preceding the suspected ARF episode (Vekemans et al., 2019). Regardless, this experience contributed to a cessation in the development of GAS vaccines for several decades, despite the mechanism of the adverse events not ever being elucidated. Now that coordinated global efforts towards a GAS vaccine are gaining momentum, the WHO have highlighted the need to understand human T cell responses to proposed GAS vaccine candidates as fundamental to defining correlates of protection (World Health Organization, 2018).

A number of different vaccine candidates are at various stages of pre-clinical and early clinical development. All current candidates are subunit vaccines, to mitigate the risks of inadvertently inducing autoimmune complications. A variety of GAS antigens are represented, some of which include the M protein. M protein is an immunodominant GAS surface protein and is thought to be a key target of humoral immunity. A 30-valent M protein mRNA vaccine has been shown to be immunogenic in animal models and Phase I clinical trials by inducing GAS-specific binding antibody responses and opsonophagocytic killing activity (Finn et al., 2024). T cell responses to GAS vaccine antigens remain almost entirely unexplored. A notable exception is the Combo# 5 vaccine, a multicomponent mRNA lipid-nanoparticle non-M protein GAS vaccine, which induced splenic CD4+ and CD8+ T cell proinflammatory cytokine responses and increased Tfh number (Harbison-Price et al., 2025). However, the antigen-specificity of these T cell responses was not reported. Glycan-specific B cell responses can be driven by T cells, and passive immunisation of monoclonal antibodies (mAbs) against GAS bacterial glycans has been identified as a promising therapeutic (Fryer et al., 2026; Temming et al., 2025). In sum, GAS vaccine studies would benefit from a standardised approach to measuring clinically important T cell responses.

In mice, GAS vaccine adjuvant choice has been shown to influence the balance of Th1/Th2 polarisation and subsequent protection from infection. Adjuvants that promoted a Th1 response with IFN-γ (compared with a Th2-dominant response) were associated with protection from invasive disease, independently from antibody response (Rivera-Hernandez et al., 2020). This highlights the importance of not just T cell antigen discovery but also T cell adjuvant development for future vaccine studies.

## 8 Conclusion

We highlight the dual protective and pathogenic roles of T cells in GAS infection. T cell immunodeficiencies do not appear to be associated with increased risk of GAS infections. However, the kinetics of age-related changes in T cell reactivity and repertoire appear to map onto age-related changes in GAS epidemiology, although this association has not yet been explored experimentally. A variety of T cell functions may be protective (e.g. Th17 immunity) or alternatively may be subverted by GAS (e.g. CD8+ T cells). A key subversion mechanism involves superantigen, which could exploit the relatively more mature T cell functionality observed in school-aged children, compared with infants. Future research priorities should focus on identifying, refining and validating clinically protective T cell subsets/functions alongside the adjuvants and GAS antigens that are capable of inducing them. Given that current mechanistic evidence is derived mainly from murine studies, these future studies should be performed and validated in human experimental models such as observation of natural infection in field studies, *in vitro* human systems (e.g. organoids) and human challenge. This review has brought together some preliminary evidence that T cells may contribute to protection from GAS, however careful consideration will be required to overcome the barriers in translating these ideas to human immunity and vaccines.

## Group members of iSpy-LIFE network collaborators

Barnett, T., Telethon Kids Institute; Darboe, S., MRC Unit The Gambia at The London School of Hygiene and Tropical Medicine; De Silva, T., MRC Unit The Gambia at The London School of Hygiene and Tropical Medicine; Engel, M., The University of Cape Town; Finn, A., University of Bristol; Fulurija, A., Telethon Kids Institute; Goenka, A., University of Bristol; Halliday, A., University of Bristol; Klenerman, P., University of Oxford; Malmstrom, J., Lund University; Marks, M., London School of Hygiene and Tropical Medicine; McCormick, J., The University of Western Ontario; Okomo, U., MRC Unit The Gambia at The London School of Hygiene and Tropical Medicine; Parks, T., Imperial College London; Sriskandan, S., Imperial College London; Van Sorge, N., Amsterdam University Medical Centers; Zuhlke, L. The University of Cape Town.
